# The utility of PacBio circular consensus sequencing for characterizing complex gene families in non-model organisms

**DOI:** 10.1186/1471-2164-15-720

**Published:** 2014-08-26

**Authors:** Peter A Larsen, Amy M Heilman, Anne D Yoder

**Affiliations:** Department of Biology, Duke University, Durham, NC 27708 USA; Primatology Department, Max Planck Institute for Evolutionary Anthropology, Leipzig, 04103 Germany

**Keywords:** Chemosensory genes, *Microcebus murinus*, Multigene family, Pacific Biosciences, Pheromone detection, Single molecule real-time sequencing

## Abstract

**Background:**

Molecular characterization of highly diverse gene families can be time consuming, expensive, and difficult, especially when considering the potential for relatively large numbers of paralogs and/or pseudogenes. Here we investigate the utility of Pacific Biosciences single molecule real-time (SMRT) circular consensus sequencing (CCS) as an alternative to traditional cloning and Sanger sequencing PCR amplicons for gene family characterization. We target vomeronasal gene receptors, one of the most diverse gene families in mammals, with the goal of better understanding intra-specific V1R diversity of the gray mouse lemur (*Microcebus murinus*). Our study compares intragenomic variation for two V1R subfamilies found in the mouse lemur. Specifically, we compare gene copy variation within and between two individuals of *M. murinus* as characterized by different methods for nucleotide sequencing. By including the same individual animal from which the *M. murinus* draft genome was derived, we are able to cross-validate gene copy estimates from Sanger sequencing versus CCS methods.

**Results:**

We generated 34,088 high quality circular consensus sequences of two diverse V1R subfamilies (here referred to as V1R*I* and V1R*IX*) from two individuals of *Microcebus murinus*. Using a minimum threshold of 7× coverage, we recovered approximately 90% of V1R*I* sequences previously identified in the draft *M. murinus* genome (59% being identical at all nucleotide positions). When low coverage sequences were considered (i.e. < 7× coverage) 100% of V1R*I* sequences identified in the draft genome were recovered. At least 13 putatively novel V1R loci were also identified using CCS technology.

**Conclusions:**

Recent upgrades to the Pacific Biosciences *RS* instrument have improved the CCS technology and offer an alternative to traditional sequencing approaches. Our results suggest that the *Microcebus murinus* V1R repertoire has been underestimated in the draft genome. In addition to providing an improved understanding of V1R diversity in the mouse lemur, this study demonstrates the utility of CCS technology for characterizing complex regions of the genome. We anticipate that long-read sequencing technologies such as PacBio SMRT will allow for the assembly of multigene family clusters and serve to more accurately characterize patterns of gene copy variation in large gene families, thus revealing novel micro-evolutionary patterns within non-model organisms.

**Electronic supplementary material:**

The online version of this article (doi:10.1186/1471-2164-15-720) contains supplementary material, which is available to authorized users.

## Background

Multigene families have played a fundamental role in the evolution of metazoan genomes [1–5]. Mechanisms such as gene duplication, gene conversion, and lineage diversification underlie multigene family complexity and contribute to genetic patterns that can be extremely difficult to molecularly characterize [6, 7]. Whereas processes such as positive selection and lineage diversification can yield gene copies of increasing nucleotide divergence, opposing processes such as gene duplication and gene conversion can yield copies that are so similar that they are virtually impossible to distinguish from sequencing error [8, 9]. The accurate characterization of gene copy number is fundamental to the differentiation of paralogy and orthology, and by extension, to the identification of heterozygotes versus homozygotes. This latter distinction is in turn central to determining the effects of genotype on phenotype, with the MHC (Major Histocompatibility Complex) gene family offering a classic example [10–13].

Given the intrinsic interest of accurate gene copy representation, it follows that methods of molecular characterization should be highly sensitive both to levels of low nucleotide diversity and to regions of high complexity. Unfortunately, such is not presently the case for organisms that lack a well-characterized genome: i.e., non-model organisms. Although low-coverage “draft” genomes are increasingly available for non-model organisms, these draft genomes are notoriously unreliable for accurate gene calling, particularly for regions of high genomic complexity [6, 14]. Thus, until such time that high-coverage, fully-assembled and annotated genomes are available for all species of interest, alternative molecular methods are desirable.

**Figure 1:**
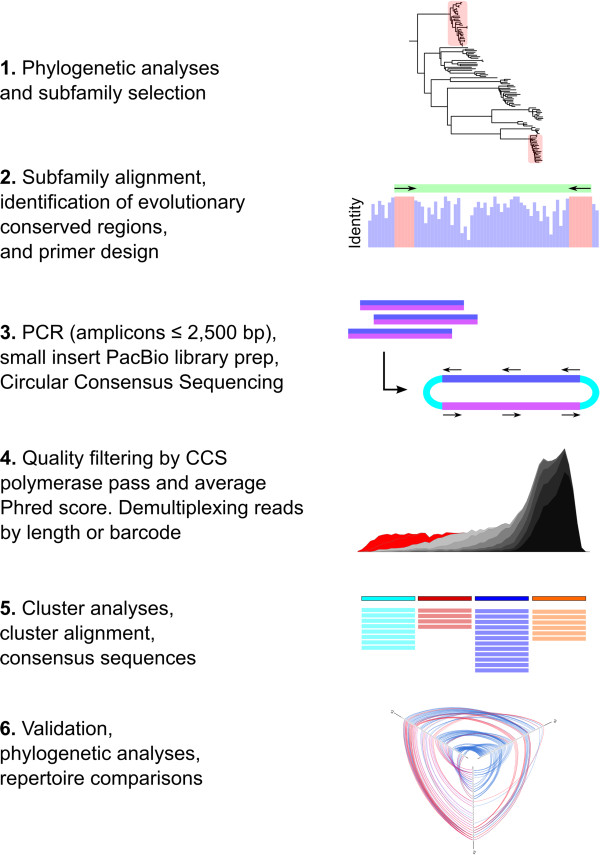
**Experimental design for characterization of V1R diversity using PacBio circular consensus sequencing. 1)** Phylogenetic analyses of existing data are performed to identify and select clades of closely related sequences (i.e. subfamilies). **2)** Individual subfamilies are aligned and evolutionary conserved regions are identified for PCR primer design. **3)** Double stranded PCR amplicons are used for library preparation and circular consensus sequencing is performed. **4)** CCS sequences are filtered based on CCS pass and average Phred score. Sequences are demultiplexed based on length, phylogenetic clustering, or barcode. **5)** Cluster analyses are performed on filtered CCS data, *de novo* chimera detection methods remove putative PCR chimeras, cluster alignments are checked for accuracy, and consensus sequences are generated. **6)** Consensus sequences are validated based on comparisons across individuals or with existing sequence data.

Long-read next generation sequencing technologies provide unique opportunities for genome-based research of non-model organisms [15]. Recent upgrades to the Pacific Biosciences *RS* instrument have improved sequence accuracy of circular consensus sequencing (CCS) yielding high-quality sequences about 500 to ~2,500 base pairs in length. CCS allows for the repeated sequencing of individual inserts and, depending on template length, stochastic sequencing errors are reduced with each CCS pass [16, 17]. Here, we investigate the utility of SMRT CCS as an alternative to traditional approaches to sequencing large closely related gene families (e.g. Sanger sequencing of cloned products). We used SMRT CCS technology, in combination with clustering algorithms and phylogenetic analyses, to measure subfamily gene diversity of vomeronasal G protein-linked receptors within a non-model primate, the gray mouse lemur (*Microcebus murinus*; Figure 1). This species was selected for analysis for several reasons. First, recent work has shown that the V1R complex is extremely diverse with a very high proportion of intact gene copies in this primate, perhaps having the highest proportion of functional V1R copies of any mammal [18]. Second, a draft genome is available for comparison against which we can validate the data generated by our study. And third, a deep-coverage fully-assembled genome is expected for release within the coming year (J. Rogers, pers. com.) allowing for final validation of gene copies generated from more approximate methods (i.e., bioinformatic mining of the draft genome).

The vomeronasal organ (VNO) is the primary olfactory organ responsible for pheromone detection in mammals and two families of VNO G protein-linked receptors (V1R and V2R) allow for the recognition of different classes of chemosensory cues. V1Rs are typically encoded by a single coding exon (~900-1000 base pairs) and are distantly related to bitter taste receptors whereas V2Rs are encoded by multiple coding exons and are closely related to Ca^2+^-sensing receptors [19–21]. Much research has been directed to distinguishing V1R receptors, owing to their single coding exon and relatively short sequence length characteristics [18, 20, 22]. However, despite recent advancements in the understanding of V1R sequence diversity in mammals [18, 23, 24], relatively little is known about the intra-specific or intra-individual (i.e., intragenomic) V1R diversity of many non-model species. This is because V1R repertories are hypothesized to evolve rapidly and are likely lineage-specific, resulting in relatively few one-to-one orthologs between species [18, 25]. These factors make characterization of the V1R repertoire difficult for DNA sequence based studies because i) traditional approaches to sequencing large, closely related gene families can be time consuming and expensive (e.g. cloning and subsequent Sanger sequencing of PCR amplicons), and ii) short-read high-throughput sequence data of such gene families are difficult to assemble given high sequence similarity and the potential for multiple paralogs to exist throughout the genome.

Many prosimian species (e.g. bushbabies, lemurs, lorises, and tarsiers) have evolved highly specialized pheromone communication mechanisms and are hypothesized to have a large number of functional V1R genes [18, 26–29]. In particular, mouse lemurs practice complex chemosensory communication (e.g. urine washing, scent marking, etc.) and accordingly, species of mouse lemurs have diverse V1R repertoires [18, 29, 30]. We present SMRT CCS data from two V1R subfamilies of two individuals of the gray mouse lemur (*Microcebus murinus*). Our study is designed to make comparisons of a biological nature (i.e., intragenomic versus intraspecific sequence variation) and of a methodological nature (i.e., Sanger sequencing of PCR clones versus CCS versus bioinformatic mining of a low-coverage draft genome; Table 1). In order to make these comparisons, we generated sequences via CCS of the same individual *M. murinus* from which the draft genome was generated/assembled (hereafter referred to as *M. murinus* 1). A second individual was included to enable comparisons within a single genome versus comparisons between genomes within a single species. This second individual was also used in a previous study of strepsirrhine V1R variation that utilized Sanger sequencing of cloned amplicons [29] (hereafter referred to as *M. murinus* 2). In summary, our study design allows for comparison of sequence characterization of a complex multigene family derived from distinct methods and sequencing technologies (Table 1).

**Table 1:** **Intra- and intergenomic comparisons of** ***Microcebus murinus*** **V1R subfamily diversity examined herein**

*Intragenomic*	
***M. murinus*** **1**	
V1R*I*	
	CCS vs. draft genome^1^
V1R*IX*	
	CCS vs. draft genome^1^
***M. murinus*** **2**	
V1R*I*	
	CCS vs. Sanger^2^
*Intergenomic*	
***M. murinus*** **1** ***vs. M. murinus*** **2**	
V1R*I*	
	CCS vs. CCS
	draft genome^1^ vs. CCS
	draft genome^1^ vs. Sanger^2^
V1R*IX*	
	CCS vs. CCS
	draft genome^1^ vs. CCS

CCS = PacBio Circular Consensus Sequencing. V1R subfamily nomenclature (V1R*I* and V1R*IX*) follows Hohenbrink *et al*. [30].

^1^V1R sequences mined by Young *et al.* [18].

^2^V1R*I* sequences originating from Sanger sequencing of cloned amplicons [29].

## Results

### CCS quality and clustering

**Figure 2:**
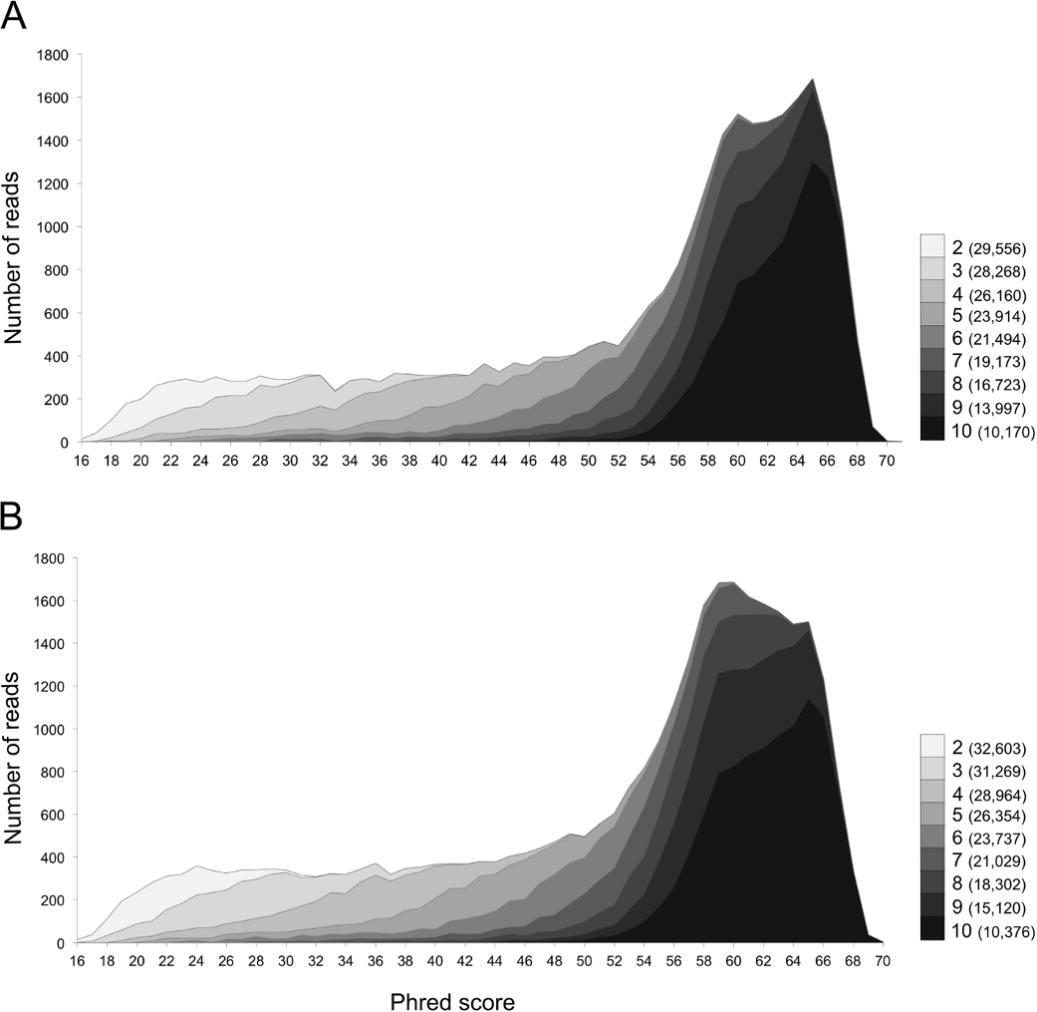
**Average read quality based on circular consensus sequencing polymerase pass.** Shading identifies CCS pass (2–10) and adjacent numbers are read count per CCS pass category. Panel **A** corresponds to SMRT Cell 1 (library 1; *M. murinus* 1) and panel **B** to SMRT Cell 2 (library 2; *M. murinus* 2).

We generated 62,159 CCS reads (minimum of 2 CCS passes; SMRT cell 1 = 29,556; SMRT cell 2 = 32,603). A bimodal distribution of sequence lengths was observed for each SMRT cell, corresponding to the V1R*I* (~725 bp) and V1R*IX* (~800 bp) amplicon sizes (Additional file 1: Figure S1). Minimum and maximum read lengths of raw data from SMRT cell 1 were 48 bp and 2,316 bp and for SMRT cell 2 min and max read lengths were 318 bp and 2,364 bp. Average quality score per read increased per CCS polymerase pass (Figure 2) and, for both libraries, we selected all reads having a minimum of 4 CCS passes and an average Phred quality score of 20 for 90% of bases per read for downstream analyses. This quality filtering approach resulted in 34,088 reads available for analysis (16,914 and 17,174 reads for *M. murinus* 1 and *M. murinus* 2, respectively; Table 2). Based on sequence length 12,625 and 11,814 reads were classified as V1R*I* and 4,289 and 5,360 reads were classified as V1R*IX* for *M. murinus* 1 and *M. murinus* 2, respectively (Table 2).

**Table 2:** **Results of CCS and cluster analyses of the V1R** ***I*** **and V1R** ***IX*** **repertoires of two individuals of** ***M. murinus***

	*M. murinus*1	*M. murinus*2
Raw CCS reads	29,739	32,837
Post quality filter	16,914	17,174
V1R*I* CCS reads	12,625	11,814
V1R*IX* CCS reads	4,289	5,360
V1R*I* Clusters (≥7×)	106	114
V1R*IX* Clusters (≥7×)	61	85
CCS V1R*I* loci (98 to 99%)	22–28	20–27
CCS V1R*IX* loci (98 to 99%)	20–24	20–28
Estimated V1R*I* repertoire	34 (30)^1^	36
Estimated V1R*IX* repertoire	32 (19)^1^	28

PacBio CCS V1R subfamily loci are estimates based on 98% to 99% genetic similarity thresholds (see Methods). Final repertoire estimates are based on cluster analyses of all available V1R*I* and V1R*IX* data (e.g. Young *et al.* [18], Yoder *et al.* [29], and this study). Chimera detection results are presented in Additional file 1: Figure S2. ^1^Number of functional genes mined from the draft *M. murinus* genome by Young *et al.* [18].

Clustering analyses resulted in 8,545 and 8,694 V1R*I* clusters and 2,936 and 3,673 V1R*IX* clusters for *M. murinus* 1 and 2, respectively (Additional file 1: Figure S2). Of these, approximately 18% and 17% of V1R*I* clusters and 5.4% and 4.6% of V1R*IX* clusters were identified as putative chimeras, *M. murinus* 1 and 2 respectively (Additional file 1: Figure S2). The majority of the chimeras (85%; n = 2,861) consisted of singleton clusters and only 13 chimeras had cluster sizes greater than a 7× threshold. Clusters consisting of putative chimeras were removed prior to all downstream analyses. Results of cluster analyses, including *de novo* chimera detection results, are presented in Table 2 and Additional file 1: Figure S2. We identified 15 clusters as consisting of putative pseudogenes and these were also excluded from downstream analyses. Final analyses were performed on consensus sequences from 106 and 114 V1R*I* clusters and 61 and 85 V1R*IX* clusters for *M. murinus* 1 and 2, respectively (≥7× coverage; Table 2). These consensus sequences were aligned using 98% and 99% similarity thresholds (see Methods) in order to determine the minimum and maximum number of V1R genes obtained using PacBio CCS technology (see Table 2). For *M. murinus* 1, 13 CCS V1R genes were not identified in sequences mined from the draft genome [18] and are considered novel.

### Comparisons between CCS and draft genome V1R sequences

**Figure 3:**
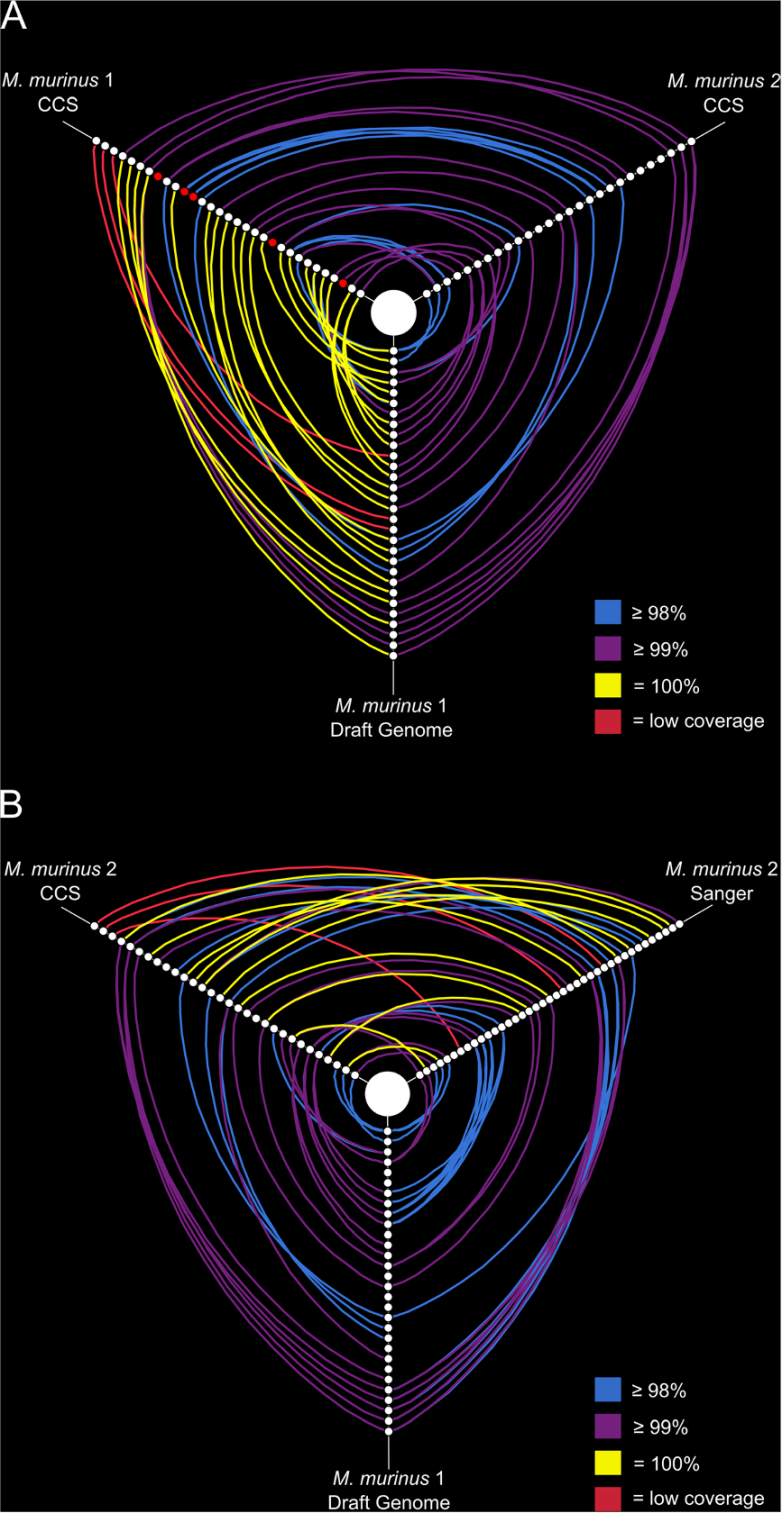
**Hive plots of sequence similarity among V1R** ***I*** **repertoires.** Distinct V1R*I* loci are displayed as nodes phylogenetically arranged along three axes. Blue, purple, and yellow arcs identify loci sharing 98%, 99%, and 100% nucleotide sequence variation, respectively. Red arcs identify loci recovered with PacBio CCS between 1× and 6× coverage. Panel **A** compares the V1R*I* repertoires of *M. murinus* 1 (CCS), *M. murinus* 2 (CCS), and draft genome sequences mined by Young *et al*. [18]. Red nodes identify loci that are unique to PacBio CCS within *M. murinus* 1. Panel **B** compares the V1R*I* repertoires of *M. murinus* 2 (CCS), *M. murinus* 2 [29], and draft genome sequences mined by Young *et al*. [18].

**Figure 4:**
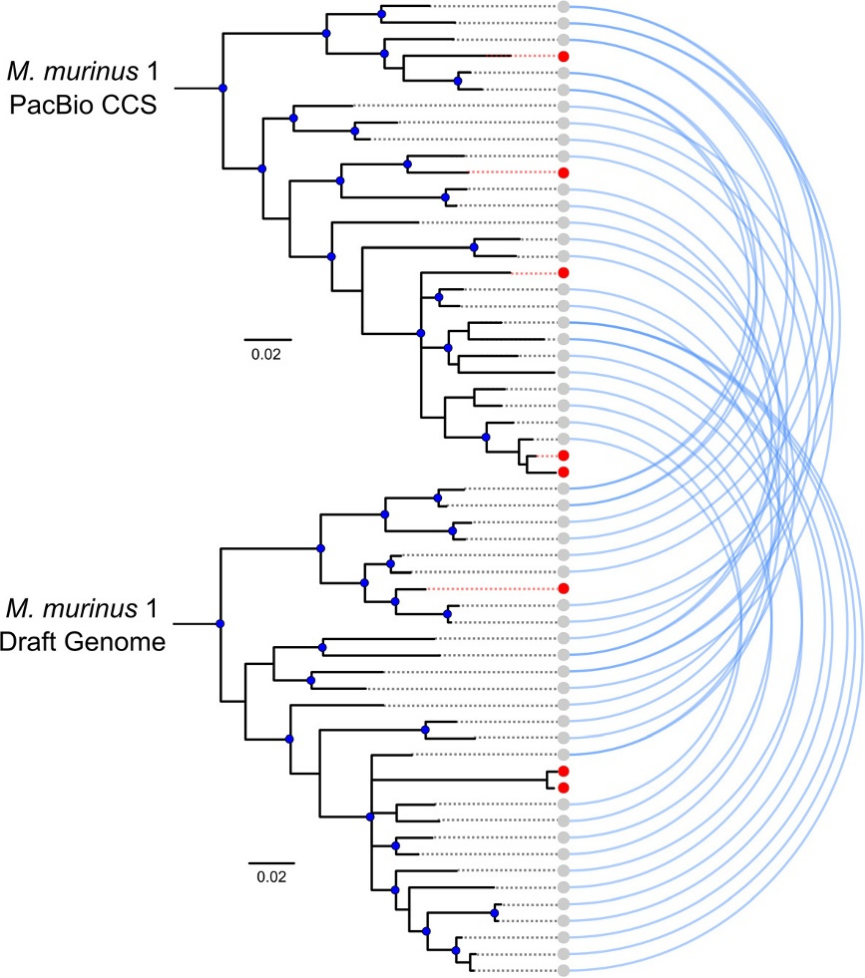
**Arcplot of CCS and draft genome V1R** ***I*** **Bayesian phylogenies.** Blue lines identify loci sharing > 98% sequence homology. Red lineages identify un-sampled or unique loci. Statistically supported clades are identified with blue nodes (BPP > 0.95 and BS > 75).

Young *et al.* [18] identified ~214 functional V1R genes within the draft *M. murinus* genome. Our *M. murinus* 1 is the same individual from which the draft *M. murinus* genome was sequenced, thus allowing for validation of our newly generated V1R CCS data. Using a minimum of 7× coverage, and a 98% similarity threshold, we recovered 27 of 30 functional V1R*I* subfamily sequences present within the *M. murinus* draft genome and 16 of these sequences were identical across all base pairs (Figures 3 and 4). When low coverage CCS reads were considered (i.e. < 7× cluster size) 100% of draft genome V1R*I* sequences were recovered (Figure 3). Of the CCS V1R*I* gene copies that we recovered, five were unique to the CCS data; i.e., they were not identified within the draft genome assembly in a previous study [18]. Collectively, the V1R*I* data (CCS sequences and those mined from the draft genome) suggest a conservative estimate of that subfamily repertoire to be 34 loci for the subfamily repertoire, a 12% increase from sequences recovered from the draft genome (Table 2). Nucleotide diversity, estimated mutation rate, and average number of nucleotide differences between V1R*I* sequences originating from CCS and those gathered from the *M. murinus* draft genome were similar (Table 3), with no significant difference being observed in the nucleotide variation between sequences generated by CCS technology and the draft genome V1R*I* repertoire (Additional file 1: Table S2).

**Table 3:** **Polymorphism statistics of DNA sequence data for the V1R subfamilies examined herein**

*M. murinus*1	*N*	Fragment size (bp)	S	*k*	π	θ _*W*_	Syn	NonSyn
V1R*I* (CCS)	29	678	223	55.75	0.083	0.084	160.78	508.22
V1R*I* (genome)	30	678	225	57.60	0.086	0.084	160.17	508.83
V1R*IX* (CCS)	24	750	199	42.22	0.056	0.071	186.69	560.31
V1R*IX* (genome)	19	750	181	42.88	0.057	0.069	186.75	563.25
***M. murinus*** **2**								
V1R*I* (CCS)	27	678	228	54.83	0.082	0.088	160.95	508.05
V1R*I* (Sanger)	43	678	271	56.96	0.085	0.093	161.01	507.99
V1R*IX* (CCS)	30	750	214	45.69	0.061	0.072	187.28	562.72

Number of sequences per methodology (*N*), number of segregating sites (S), average number of nucleotide differences between sequences (*k*), mean nucleotide diversity (π), Watterson’s estimator of mutation rate (θ_*W*_), raw number of synonymous (Syn) and nonsynonomous (NonSyn) mutations. CCS = PacBio Circular Consensus Sequencing; genome = sequences mined from the *M. murinus* draft genome [18]; Sanger = sequences originating from Yoder *et al*. [29].

**Figure 5:**
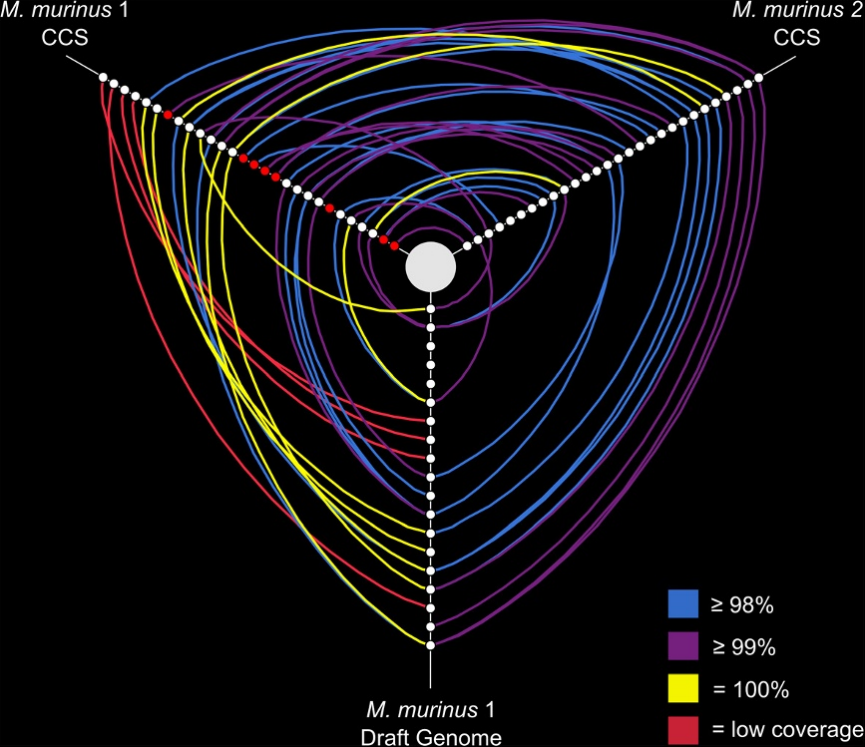
**Hive plots of sequence similarity among V1R** ***IX*** **repertoires.** Distinct V1R*IX* loci are displayed as nodes phylogenetically arranged along three axes. Blue, purple, and yellow arcs identify loci sharing 98%, 99%, and 100% nucleotide sequence variation, respectively. Red arcs identify loci recovered with PacBio CCS between 1× and 6× coverage. The V1R*IX* repertoires of *M. murinus* 1 (CCS), *M. murinus* 2 (CCS), and draft genome sequences mined by Young *et al*. [18] are compared. Red nodes identify loci that are unique to PacBio CCS within *M. murinus* 1.

We recovered approximately 58% of V1R*IX* draft genome sequences and this value increased to 79% with the inclusion of low coverage CCS reads (Figure 5). Seven V1R*IX* sequences were identical across all base pairs with sequences mined from the draft genome (Figure 5). Eight putatively novel V1R*IX* loci (i.e. absent from the draft genome assembly) were identified yielding an estimate of 32 loci for the *M. murinus* V1R*IX* subfamily repertoire, a 68% increase from those previously identified in the draft genome. Patterns of nucleotide variation were similar between the CCS V1R*IX* data and the draft genome sequences (Table 3) and no statistical difference was observed in the nucleotide variation between the two datasets (Additional file 1: Table S2).

**Figure 6:**
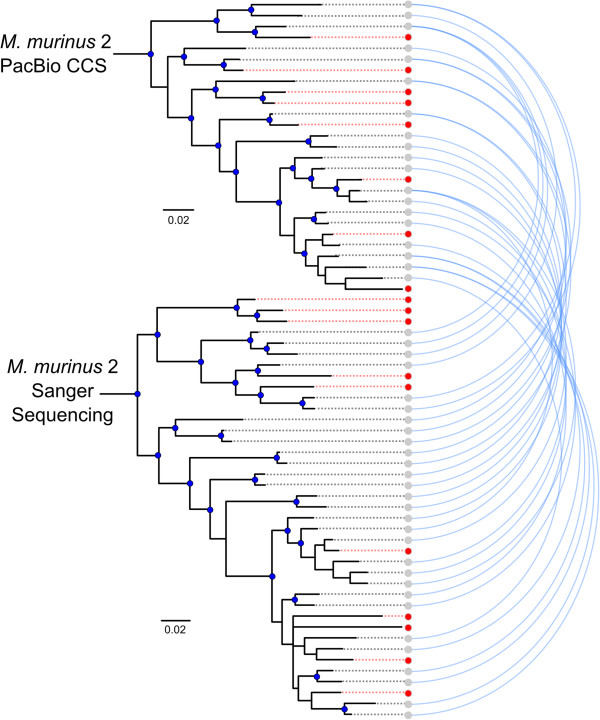
**Arcplot of CCS and Yoder** ***et al.*** **V1R** ***I*** **Bayesian phylogenies.** Blue lines identify loci sharing > 98% sequence homology. Red lineages identify un-sampled or unique loci. Statistically supported clades are identified with blue nodes (BPP > 0.95 and BS > 75).

Molecular variation of the strepsirrhine V1R*I* subfamily has been explored using a traditional Sanger sequencing of cloned inserts approach [29]. We used the same PCR primers as Yoder *et al.* [29] as well as DNA from one individual of *M. murinus* used in that study (herein *M. murinus* 2). We recovered approximately 82% of the sequences reported by Yoder *et al.* [29] for *M. murinus* 2, with 12 sequences being identical (Figures 3 and 6). We identified a maximum of 27 putative V1R*I* loci within the *M. murinus* 2 CCS data. When these data were combined with the Sanger sequence data [29] the V1R*I* repertoire size from *M. murinus* 2 is estimated to consist of ~36 loci (98% sequence similarity threshold). When compared with V1R*I* CCS sequence data, the nucleotide diversity and average number of nucleotide differences were slightly higher in sequences originating using the Sanger sequencing of clones approach [29] (Table 3), however no statistical difference was observed in nucleotide variation between the two datasets (Additional file 1: Table S2).

### *M. murinus*V1R repertoire comparisons

**Figure 7:**
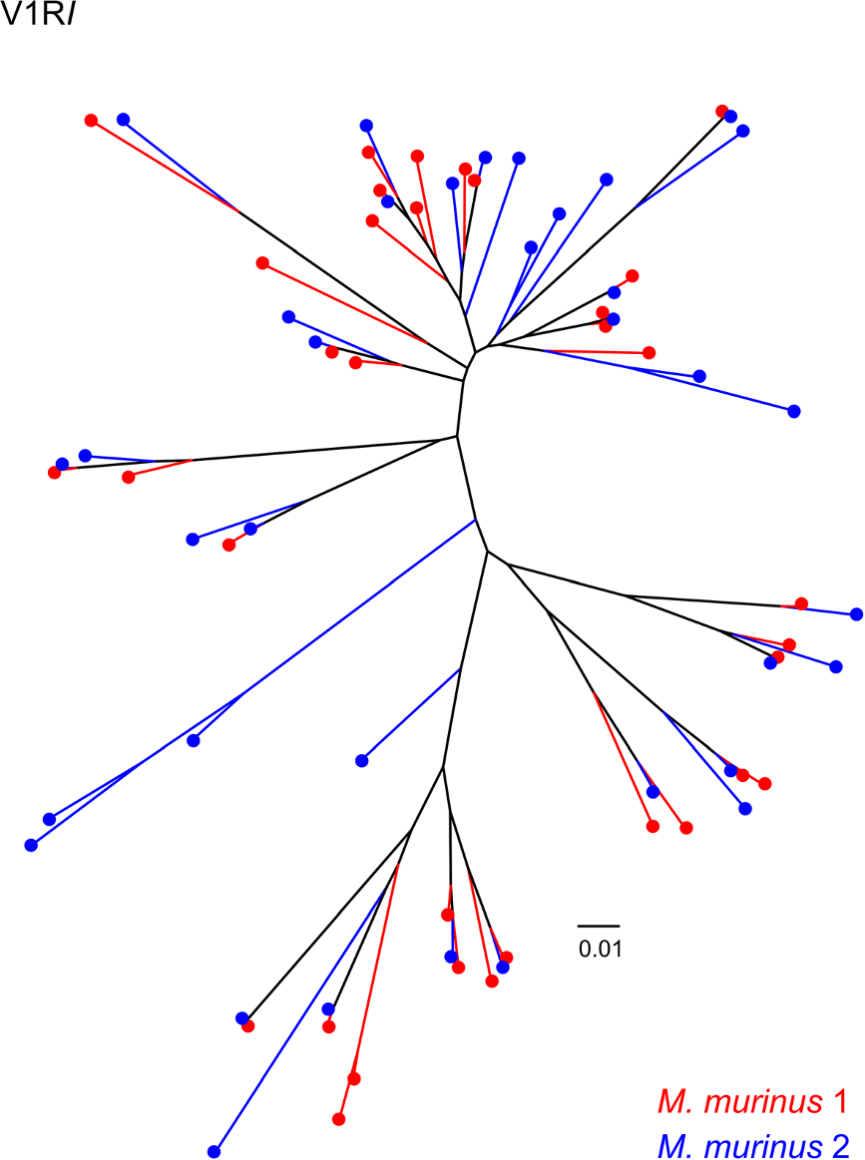
***M. murinus*** **V1R** ***I*** **repertoire.** Unrooted neighbor-joining phylogram based on amino acid sequence variation of the functional *M. murinus* V1R*I* repertoire. The phylogeny includes all known *M. murinus* V1R*I* sequences (Young *et al.*[18], Yoder *et al.*[29], and CCS data presented herein) clustered at 98% similarity threshold. Red and blue branches and terminal nodes identify individuals 1 and 2, respectively.

**Figure 8:**
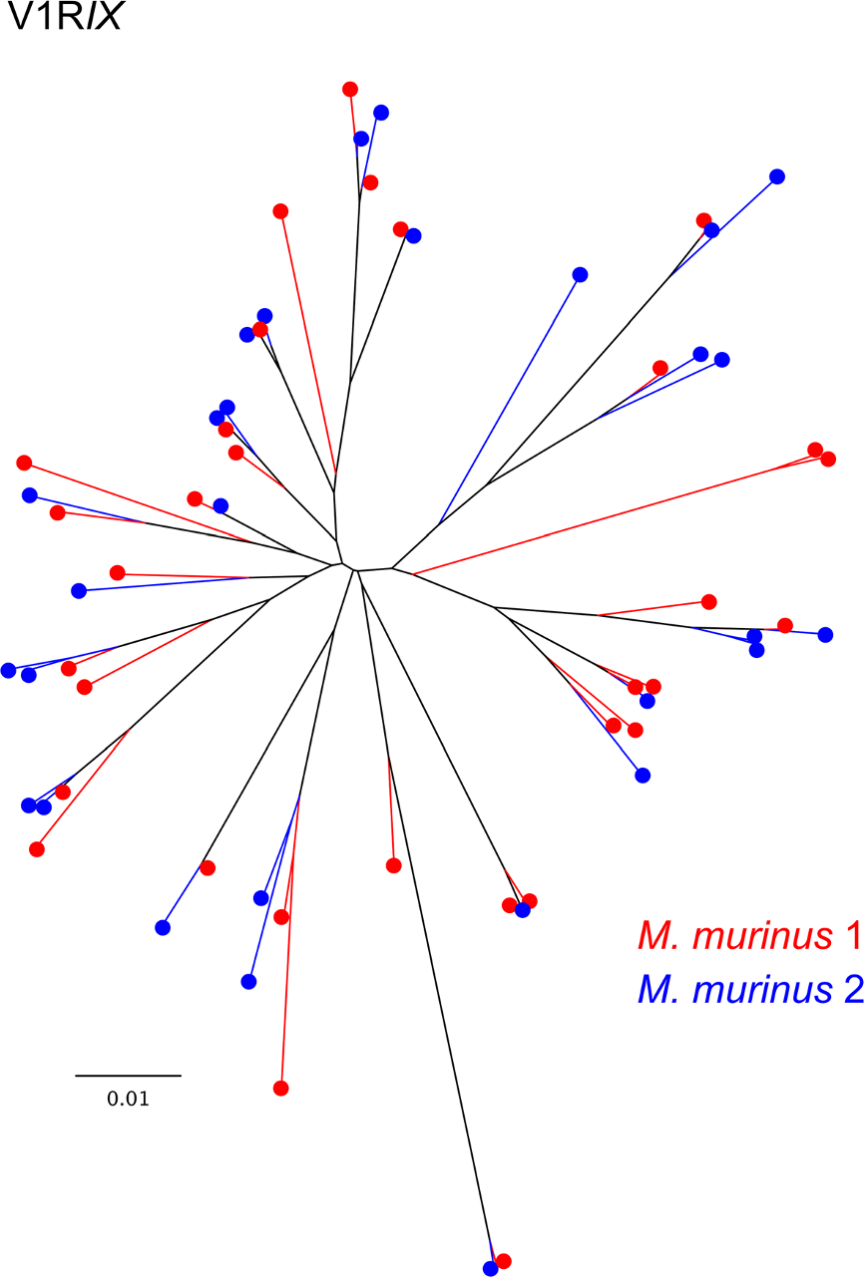
***M. murinus*** **V1R** ***IX*** **repertoire.** Unrooted neighbor-joining phylogram based on amino acid sequence variation of the functional *M. murinus* V1R*IX* repertoire. The phylogeny includes all known *M. murinus* V1R*IX* sequences (Young *et al.*[18] and CCS data presented herein) clustered at 98% similarity threshold. Red and blue branches and terminal nodes identify individuals 1 and 2, respectively.

*Microcebus murinus* 1 and 2 shared approximately 70% of their CCS V1R*I* repertoires and 15 sequences sharing greater than 99% sequence similarity were identified (Figure 3). Zero V1R*I* sequences were identified as matching across all base pairs between the two individuals (Figure 3). Alternatively, the V1R*IX* repertoires of *M. murinus* 1 and 2 overlapped by ~90% and 16 sequences were identified as sharing greater than 99% percent sequence similarity, with three of these matching at all base pairs (Figures 5 and 6). Intragenomic nucleotide distances within the V1R*I* and V1R*IX* subfamilies were 9.8% and 6.3%, respectively, for *M. murinus* 1 and values were similar for *M. murinus* 2 (Additional file 1: Table S3). All genetic distance calculations were similar for both inter- and intra-genomic comparisons and the V1R*I* subfamily exhibited greater variability relative to V1R*IX* (Additional file 1: Table S3). Phylogenetic analyses of amino acid variation of the V1R*I* and V1R*IX* repertoires for both individuals are presented in Figures 7 and 8. No significant differences in the magnitude of nucleotide variation were observed between the V1R*I* and V1R*IX* repertoires of *M. murinus* 1 and 2, respectively (Table 3).

## Discussion

### Insights into the *Microcebus murinus*V1R repertoire

Chemosensory communication has played a critical role in mammalian evolution from both physiological and behavioral perspectives. In particular, many species rely heavily on pheromones for intraspecific communication, especially with respect to sexual and social behaviors [31]. Of all mammalian orders, Primates exhibits what is perhaps the greatest variation of functional versus pseudogenized V1R diversity [18, 22, 23]. For example, no functional V1R genes have been identified in the macaque (*Macaca mulatta*) genome whereas ~214 intact V1R genes have been identified in *M. murinus* [18]. This proportion of functional versus pseudogenized V1R genes is likely correlated with a reduction of pheromone communication in some primate lineages (e.g. Old World catarrhines; [23, 32]), whereas lineages that have maintained or evolved enhanced chemical communication typically exhibit diverse V1R repertories (e.g. strepsirrhine primates [18, 29]). In particular, mouse lemurs practice complex chemosensory communications and *M. murinus* has one of the highest proportions of functional vs. pseudogenized V1R repertories of all mammalian species characterized thus far [18]. Our results reinforce this finding and suggest that the functional V1R repertoire of *M. murinus* has likely been underestimated, perhaps by as much as 25% in the V1R*I* and V1R*IX* subfamilies, collectively. This observation, coupled with recent documentation of strong positive selection throughout the mouse lemur V1R repertoire [30], strengthens hypotheses regarding the highly specialized pheromone communication mechanisms used by species of lemurs [26–29].

While the putative function of the V1R*I* subfamily is unknown [29, 30], the available data indicate that this subfamily binds a diverse variety of ligands [29]. Alternatively, the genetic variation within the V1R*IX* subfamily suggests this subfamily is more conserved, perhaps binding to a reduced number of ligand classes (Table 3; Figure 8). This hypothesis is reinforced by the observation that the two individuals examined herein share ~84% of their V1R*IX* repertoires, compared to ~60% shared V1R*I* loci, at the 98% sequence similarity threshold. Moreover, we identified three V1R*IX* loci that were identical between *M. murinus* 1 and 2, whereas zero V1R*I* loci were identical between the two individuals. These results support previous studies that have hypothesized differing rates of evolution within lemurid V1R subfamilies [29, 30]. Based on comparisons with putative mouse orthologs, Hohenbrink *et al*. [30] hypothesized that the V1R*IX* subfamily was closely related to the mouse V1Rc subfamily, a subfamily that has been shown to detect female, heterospecific, and predator cues in mice [33]. Future studies focused on the identification of the ligands associated with the nine known V1R subfamilies present in *M. murinus* will be an important advance for understanding the functional roles of these gene families and whether or not genetic variation underlying V1R repertoires contribute to the maintenance of species boundaries within the genus.

### Utility of CCS for gene family characterization and discovery in non-model species

Sanger sequencing of cloned inserts is a well-established and common approach for characterizing multigene family diversity [29, 34–36]. Although effective, this method can be time-consuming, labor-intensive, and expensive. A growing number of studies utilize next generation sequencing technologies for targeted approaches to gene validation and discovery [37, 38]. These technologies have limitations however, and issues such as systematic and stochastic error rates as well as average read lengths must be considered when developing experimental designs [6]. We selected Pacific Biosciences SMRT sequencing technology because the long read lengths eliminated the necessity for downstream assembly of highly similar fragments and because CCS reduces stochastic error rates. Moreover, the option of filtering reads by number of CCS pass provides greater flexibility to quality control of PacBio sequence data. Recent advancements to the PacBio *RS* sequencing instrument and sequencing chemistry have improved read length and accuracy [15] and, as of this writing, Pacific Biosciences has released the *RS II* upgrade which allows for higher throughput and even greater read accuracy.

Relatively few studies have utilized PacBio CCS for targeted sequencing in non-model species [39, 40]. In the absence of high quality genome assemblies, the long read lengths provided by SMRT CCS offer new opportunities for characterizing complex multigene families (e.g. immunoglobulin, MHC, olfactory receptors, V1R, etc.). The observation that our clustering approach of CCS reads resulted in capturing 100% of V1R*I* sequences mined from the draft genome assembly (including low coverage clusters), coupled with the identification of 13 putatively novel genes (Figures 3, 4, 5, 6, 7 and 8), documents that the methods reported herein are useful for gene discovery and for describing the diversity of large gene families. Moreover, no bias was detected in the nucleotide variation of sequences originating from CCS clusters with respect to those mined from the draft genome [18] or generated via Sanger sequencing of cloned inserts [29] (Table 3; Additional file 1: Table S2). Our results concerning the reduced coverage of the V1R*IX* subfamily (when compared to V1R*I*) likely stem from PCR amplification bias and/or preferential sequencing of the shorter V1R*I* sequences in pooled sequencing libraries. This finding, in addition to the identification of putative PCR chimeras by *de novo* chimera detection software (Additional file 1: Figure S2), generally agrees with other studies that have identified PCR bias and PCR artifacts within data originating from high-throughput sequencing of PCR amplicons [41].

Although our experimental design is useful for identifying potentially unrecognized gene diversity, a major drawback is the inability to distinguish closely related paralogs and to reliably identify orthologs among individuals. This problem is compounded by the observation that V1Rs are encoded by a single exon [21, 23] and therefore lack intronic sequences that may help to identify orthologs and/or paralogs. Thus, future studies aimed at characterizing V1R gene diversity in non-model species may benefit from other methods such as the targeted capture and sequencing of genomic regions harboring V1R genes (e.g., using biotinylated probes in combination with PacBio sequencing of long templates). Such an approach would be useful for identifying orthologous and paralogous genes and for characterizing allelic variation in multigene families of non-model species.

## Conclusions

Our findings suggest that the V1R repertoire of *M. murinus* is larger than previously hypothesized and underscore previous observations that low coverage genome assemblies provide a limited view of multigene-family diversity [14, 18]. Even so, it is probable that we have still underestimated V1R diversity given the potential for the clustering of closely related paralogs (i.e. < 2% sequence divergence). Importantly, the forthcoming availability of a high coverage (~150×) *M. murinus* genome (Human Genome Sequencing Center, Baylor College of Medicine) will allow our hypotheses regarding the V1R repertoire size to be more definitively tested.

Pacific Biosciences SMRT CCS provides an alternative to traditional Sanger sequencing of cloned inserts. We anticipate that the methods described herein will be useful for the characterization of diverse gene families in other non-model species where genome sequences are unavailable or consist of low coverage draft assemblies. Our results concerning the presence of putative PCR artifacts agree with previous observations [41] and necessitate the implementation of strict quality control measures when high-throughput sequencing is performed on libraries constructed from PCR amplicons. Modifications to our approach, such as barcoding and advanced targeted capture methodologies will be useful for increasing sample size and gene discovery. These methods will greatly advance genome assembly and annotation of multigene families in non-model species.

## Methods

### Molecular methods

We examined V1R sequences mined from the draft *Microcebus murinus* genome by Young *et al.* [18] and selected two diverse subfamilies (V1R*I* and V1R*IX sensu* Hohenbrink *et al.* [30]) for circular consensus sequencing (Additional file 1: Figure S3). These subfamilies were amplified from whole genomic DNA, isolated from two non-related individuals of *M. murinus*, using primers targeting conserved transmembrane regions 2 and 7 (V1R*I*) and 1 and 7 (V1R*IX*; Additional file 1: Table S1). We refer to the individual from which the draft genome was derived as *M. murinus* 1, and the second individual, included in the study by Yoder *et al.* [29], is referred to as *M. murinus* 2 (Duke Lemur Center voucher number 7013). Animal procedures were reviewed and approved by the Duke University Institutional Animal Care and Use Committee under protocol number A250-12-09.

Amplicons were obtained using a high fidelity Taq DNA polymerase (Platinum Taq; Invitrogen) and PCRs were conducted in 50 ul reactions with the following final concentrations: 1× high fidelity buffer, 2 mM MgCl2, 200 uM dNTPs, 0.8uM primers, 0.625 units Taq, and ~15 ng DNA template. The following touchdown thermal profile was used for all amplifications: initial denaturation 95°C for 3 min followed by 15 cycles of 95°C for 1 min, 60°C (1°C decrease per cycle) for 1 min, 72°C for 1 min 30 sec, then another 20 cycles of 95°C for 1 min, 45°C for 1 min, 72°C for 1 min 30 sec, and a final extension of 72°C for 10 min. PCR products were visualized on a 2% agarose gel using SYBR Green I (Lonza Rockland, Inc.) and bands within the expected size ranges (V1R*I* = ~725 bp and V1R*IX* = ~800 bp) were excised and extracted using the Mo Bio gel purification kit (Mo Bio Laboratories, Inc.).

Three PCR reactions per individual per locus were pooled separately and quantified using a NanoDrop spectrophotometer (Thermo Scientific). V1R*I* and V1R*IX* amplicons were then pooled (1.5 μg V1R*I* and 1.0 μg V1R*IX*) resulting in two 2.5 μg samples for the construction of two sequencing libraries. V1R*I* amplicons were enriched to ensure sequence coverage given the increased variation observed within the V1R*I* subfamily when compared to V1R*IX* [18, 30]. Samples were submitted to the Duke IGSP Genome Sequencing & Analysis Core Resource for real-time circular consensus sequencing using a Pacific Biosciences *RS* instrument and C2 chemistry. Two small-insert libraries (one per individual) were prepared following manufacturers protocols and were sequenced using two SMRT cells (one SMRT cell per library) with 2 × 55 min movie run times. The resulting bas.h5 files were used for downstream analyses.

### Quality filtering and sequence clustering

CCS sequences were quality filtered using pbh5tools (https://github.com/ PacificBiosciences/pbh5tools) and the Galaxy platform [42–44]. The pbh5tools package was used to extract CCS fastq sequences from bas.h5 files according to minimum number of CCS pass, thus allowing for inspection of average read quality as a function of CCS pass (see Figure 2 and Results). We used FastQC software (v0.10.1; http://www.bioinformatics.babraham.ac.uk/projects) to summarize average Phred score per CCS pass category (Figure 2). Cluster analyses were performed on sequences that originated from a minimum of 4 CCS passes and within which 90% of the bases averaged a quality score ≥ Phred 20 (1% error rate). Our pooled samples consisted of amplicons separated by ~65 bp in length, thus allowing for demultiplexing V1R*I* and V1R*IX* sequences according to length.

The USEARCH software package (v6.0) [45] was used for clustering, *de novo* PCR chimera detection, and preliminary cluster alignment. The UCHIME algorithm (as implemented within USEARCH) was used to detect putative chimeric sequences with the *de novo* mode and an -abskew parameter of 2.0. Clusters containing putative chimeras were not included in downstream analyses. Quality filtered CCS sequences were clustered based on a 98% similarity threshold with the -cluster_fast option and resulting alignments of clusters containing ≥ 7 sequences (i.e., a 7× threshold) were imported into the Geneious software package (v6.1; http://www.geneious.com) re-aligned using the MAFFT (v7.017) alignment plugin and then manually edited for accuracy. We selected the 7× coverage threshold based on our chimera detection results (i.e., 0.4% of all clusters comprised of putative chimeras contained 7 or more sequences; see Results and Additional file 1: Figure S2). Cluster consensus sequences were identified as V1R using NCBI BLAST (http://blast.ncbi.nlm.nih.gov/) and V1R subfamily membership was confirmed by phylogenetic comparisons with Hohenbrink *et al.* [30].

The minimum number of distinct V1R genes for each subfamily was estimated following Rodriguez *et al.* [46] whereby cluster consensus sequences sharing greater than 98% nucleotide homology were considered identical. This approach reduced concerns of spurious results due to sequencing error and/or repertoire inflation due to paralogous loci, but at the same time it is likely to underestimate the total number of genuine V1R paralogous copies. To overcome this limitation, to some extent, we also used a 99% minimum genetic similarity threshold to estimate maximum V1R repertoire size. Moreover, 99% is the minimum genetic similarity separating V1R sequences mined from distinct regions of the draft *M. murinus* genome [18]. Cluster consensus sequences were translated into amino acids and were checked for complete open reading frame to identify putatively functional and pseudogenized loci. Final alignments for all consensus sequences are provided in Additional file 2: Dataset 1. Given the clustering approaches described above, in combination with the observation that we used primers that bound to conserved regions within the V1R exon, we anticipated that closely related paralogs would be clustered together and thus we refrained from attempting to identify allelic variation within potentially non-homologous loci.

### Phylogenetic and statistical analyses

Alignments of PacBio CCS cluster consensus sequences with V1R data from Young *et al.* [18] (Additional file 2: Dataset 1) and Yoder *et al.* [29]; [GenBank:KF272289–KF272350] were performed using MAFFT v7.017 (gap open penalty 1.53; offset value 0.123) as implemented within the Geneious software package. Sequences originating from Yoder *et al*. [29] were also clustered based on the 98% threshold described above in order to avoid the incorporation of potentially paralogous loci in the analyses presented herein (Additional file 2: Dataset 2). Phylogenetic analyses were performed using MrBayes v.3.2 [47] and RAxML v.7.7 [48]. The GTR + gamma model of substitution was used for all Bayesian and Maximum Likelihood analyses. Statistical support for nodes was evaluated using Bayesian posterior probabilities (resulting from 5 million iterations, 4 heated chains, 25%% burn-in length) and maximum likelihood bootstrap support values (percentage of 1,000 iterations). Resulting trees were edited using FigTree v1.4 software (http://tree.bio.ed.ac.uk/software/figtree/). Pairwise sequence similarity was measured using custom BLAST searches with the percent identity output option. Sequence similarity was visualized using hive plots (jHive v0.0.18; [49]) and the arcdiagram R package (https://github.com/gastonstat/arcdiagram). The software package DNAsp v5.1 [50] was used to calculate basic polymorphism statistics for each V1R subfamily including number of segregating sites (S), average number of nucleotide differences between alleles (*k*), nucleotide diversity (π), Watterson’s estimator of population mutation rate (θ_*W*_), and number of synonymous and nonsynonymous mutations. Genetic distances were calculated using the Kimura-2 parameter (nucleotide) and p-distance (amino acid) algorithms as implemented within Mega v5.2 software [51]. Genetic divergence among V1R repertoires was assessed using Chi-square statistical tests as implemented in DNAsp.

### Availability of supporting data

Final V1R consensus sequences generated by this study have been deposited in GenBank and have the following accession numbers [KF721294 - KF721403]. CCS sequence data generated from *M. murinus* 1 (origin of the *M. murinus* draft genome) are identified with a specimen-voucher number of DGM01. Additional file 2: Dataset 1 contains alignments of all CCS data used in the final analyses as well as V1R data mined from Young *et al.* [18]. Additional file 2: Dataset 2 contains filtered V1R*I* sequences originating from Yoder *et al*. [29]. Both Additional file 2: Datasets are located at http://www.labarchives.com with the following doi:10.6070/H4G73BN0.

## Electronic supplementary material

Additional file 1: Figure S1: Read lengths of raw PacBio CCS sequences for two SMRT cells. Pooled amplicons for V1R subfamilies *I* and *IX* were sequenced from two individual of *Microcebus murinus* (SMRT cells 1 [A] and 2 [B]; see Methods and Results). **Figure S2.** Results of *de novo* chimera detection analysis of CCS sequence data. Number of CCS clusters and putative chimeras for V1R*I* (A) and V1R*IX* (B) subfamily sequences from *M. murinus* 1 and 2, respectively. **Figure S3.** Neighbor-joining phylogeny of functional V1R sequences mined from the draft *M. murinus* genome. Highlighted clades were selected for CCS sequencing. Subfamily nomenclature follows Hohenbrink et al. [30]; *I–IX*. **Table S1.** Primer sequences used to amplify *Microcebus murinus* V1R*I* and V1R*IX* subfamiles. **Table S2.** Results of Chi-squared tests of genetic differentiation. **Table S3.** Intra- and inter- V1R subfamily genetic distances (percentages) for *M. murinus*. (PDF 1 MB)

Additional file 2: **Supplementary Data.** (TXT 141 KB)

## Abbreviations

CCS: Circular consensus sequencing
V1R: Vomeronasal type 1 receptor
V2R: Vomeronasal type 2 receptor
SMRT: Single molecule real-time.
