# Supplementary material for: The utility of PacBio circular consensus sequencing for characterizing complex gene families in non-model organisms
Source: BMC Genomics. 2014 Aug 26;15(1):720. doi: 10.1186/1471-2164-15-720 (PMC4152597; doi:10.1186/1471-2164-15-720)
Supplement: Supplementary file 1 — Additional file 1: Figure S1: Read lengths of raw PacBio CCS sequences for two SMRT cells. Pooled amplicons for V1R subfamilies I and IX were sequenced from two individual of Microcebus murinus (SMRT cells 1 [A] and 2 [B]; see Methods and Results). Figure S2. Results of de novo chimera detection analysis of CCS sequence data. Number of CCS clusters and putative chimeras for V1RI (A) and V1RIX (B) subfamily sequences from M. murinus 1 and 2, respectively. Figure S3. Neighbor-joining phylogeny of functional V1R sequences mined from the draft M. murinus genome. Highlighted clades were selected for CCS sequencing. Subfamily nomenclature follows Hohenbrink et al. [30]; I–IX. Table S1. Primer sequences used to amplify Microcebus murinus V1RI and V1RIX subfamiles. Table S2. Results of Chi-squared tests of genetic differentiation. Table S3. Intra- and inter- V1R subfamily genetic distances (percentages) for M. murinus. (PDF 1 MB) [file 12864_2013_6387_MOESM1_ESM.pdf]

Supplementary Material

Larsen et al.
Utility of PacBio CCS for characterizing multigene family diversity

Supplementary Data
Supplementary Datasets are located at www.labarchives.com with the following DOI: 10.6070/H4G73BN0

Supplementary Figures

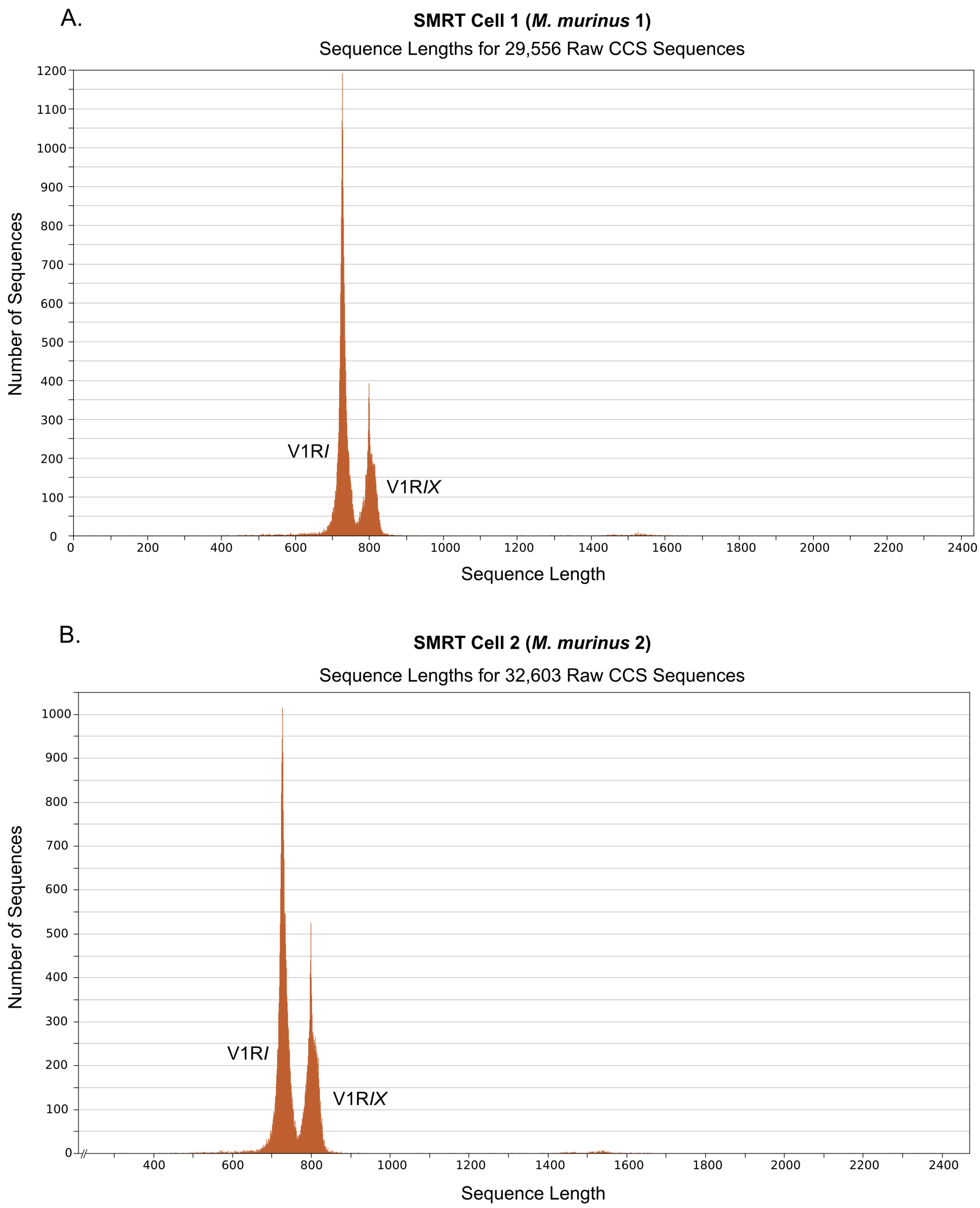

Supplementary Figure 1. Read lengths of raw PacBio CCS sequences for two SMRT cells. Pooled amplicons for V1R subfamilies *I* and *IX* were sequenced from two individuals of *Microcebus murinus* (SMRT cells 1 [**A**] and 2 [**B**]; see Methods and Results).

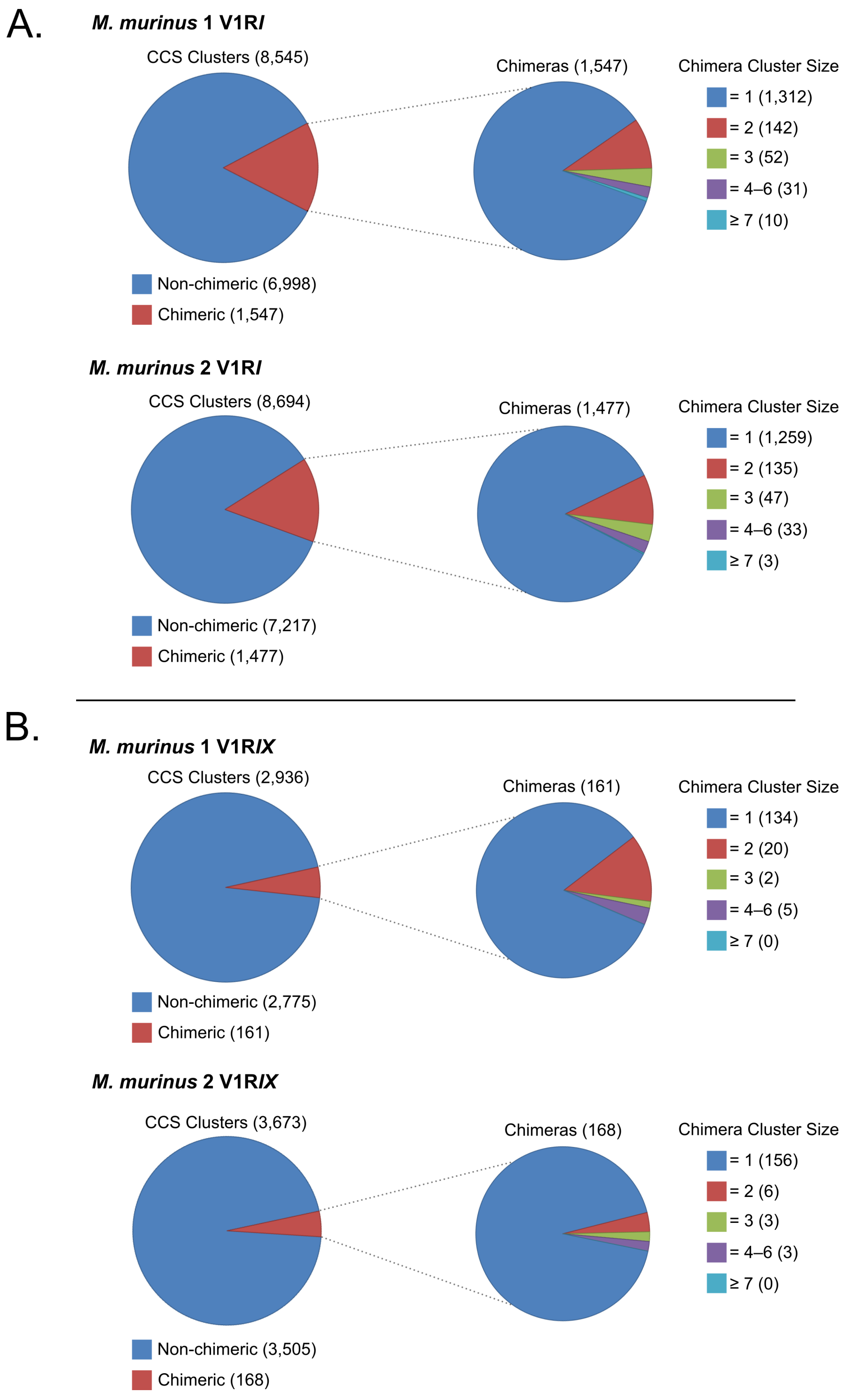

Supplementary Figure 2. Results of *de novo* chimera detection analysis of CCS sequence data. Number of CCS clusters and putative chimeras for V1R*I* (**A**) and V1R*IX* (**B**) subfamily sequences from *M. murinus* 1 and 2, respectively.

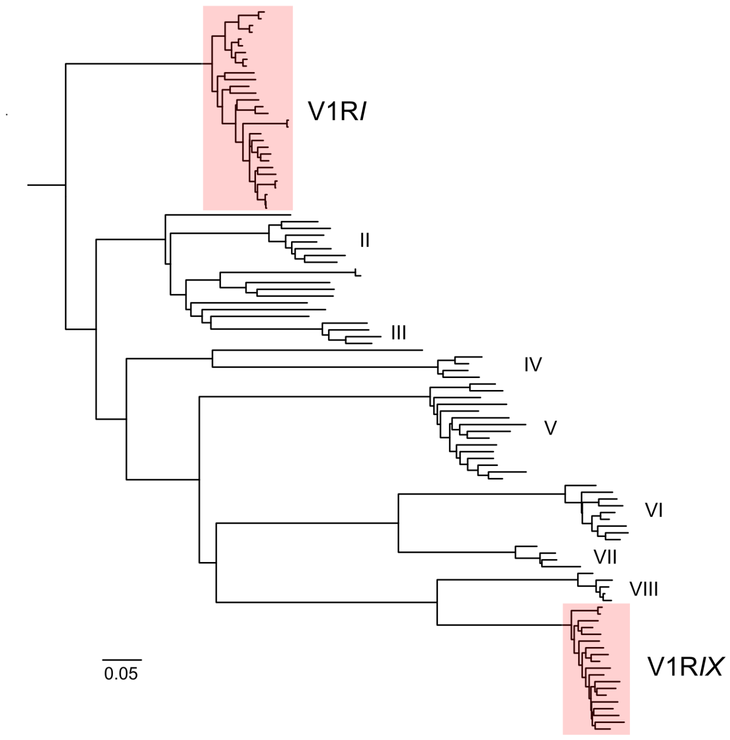

Supplementary Figure 3. Neighbor-joining phylogeny of functional V1R sequences mined from the draft *M. murinus* genome. Highlighted clades were selected for CCS sequencing. Subfamily nomenclature follows Hohenbrink *et al*. (2012; *I–IX*).

Supplementary Tables

Supplementary Table 1. Primer sequences used to amplify *Microcebus murinus* V1R*I* and V1R*IX* subfamiles.

Primer	V1R Subfamily	Orientation	Primer Sequence	Source
V1RG1F	V1R*I*	Forward	CTC AAC CAG CTG GTC TTA GCY AAC	Yoder et al. 2014
V1RG1R	V1R*I*	Reverse	GAC AAT GAA CAC AAA GGG GCT GAA	Yoder et al. 2014
MMV1RIXF	V1R*IX*	Forward	CAY AGC CAA YAY CTT	This paper
MMV1RIXR	V1R*IX*	Reverse	ATT ATC ACH AAA GGA CTY A	This paper

Supplementary Table 2. Results of Chi-squared tests of genetic differentiation.

M. murinus 1	χ^2	***P***	**d.f.**
V1R*I* CCS vs. genome	36	0.879	47
V1R*IX* CCS vs. genome	32.86	0.663	37
M. murinus 2			
V1R*I* CCS vs. Sanger	59.44	0.638	64
**Final repertoire comparisons**			
V1R*I* *M. murinus* 1 vs. *M. murinus* 2	88	0.450	87
V1R*IX* *M. murinus* 1 vs. *M. murinus* 2	58.99	0.470	59

Supplementary Table 3. Intra- and inter- V1R subfamily genetic distances (percentages) for *M. murinus*.

Intra-individual	Nucleotide	Amino Acid
M. murinus 1 (V1R*I*)	9.6	14.4
M. murinus 2 (V1R*I*)	9.8	14.8
M. murinus 1 (V1R*IX*)	6.3	11.2
M. murinus 2 (V1R*IX*)	6.4	10.8
Inter-individual		
M. murinus 1 vs. 2 (V1R*I*)	9.6	14.4
M. murinus 1 vs. 2 (V1R*IX*)	6.4	10.8

Hohenbrink P, Radespiel U, Mundy NI: **Pervasive and ongoing positive selection in the vomeronasal-1 receptor (V1R) repertoire of mouse lemurs.** *Molecular biology and evolution* 2012, **29:**3807-3816.

Yoder A, Chan L, dos Reis M, Larsen P, Campbell C, Rasolarison R, Barrett M, Roos C, Kappeler P, Bielawski J, Yang Z: **Molecular evolutionary characterization of a novel V1R subfamily in strepsirrhine primates.** *Genome biology and evolution* 2014, **6:**213-227.

Young JM, Massa HF, Hsu L, Trask BJ: **Extreme variability among mammalian V1R gene families.** *Genome research* 2010, **20:**10-18.
